# Low‐grade osteosarcoma is predominant in gnathic osteosarcomas: A report of seven cases of osteosarcoma of the jaw

**DOI:** 10.1002/cre2.442

**Published:** 2021-05-19

**Authors:** Aya Sasaki, Hidetaka Miyashita, Miho Kawaida, Kaori Kameyama

**Affiliations:** ^1^ Department of Pathology and Laboratory Medicine Tokyo Dental College Ichikawa General Hospital Ichikawa Japan; ^2^ Division of Diagnostic Pathology Keio University Hospital Tokyo Japan; ^3^ Division of Oral and Maxillofacial Surgery, Department of Dentistry and Oral Surgery Keio University School of Medicine Tokyo Japan

**Keywords:** intramembranous ossification, jaw, low‐grade, osteosarcoma

## Abstract

**Objective:**

Primary osteosarcoma of the jaw bones is very rare, and histological features of gnathic osteosarcoma remain obscure. The purpose of this study was to describe the clinicopathological features of gnathic osteosarcoma.

**Materials and methods:**

Seven cases of gnathic osteosarcoma from Japan diagnosed during the period between 2000 and 2016 were examined retrospectively. The histology of the surgical pathology materials was reviewed by two pathologists. Clinical information was obtained from the hospital's information system.

**Results:**

Of the seven cases, two patients had secondary osteosarcomas. As for the five cases of primary osteosarcoma, their ages ranged from 26 to 58 years (mean: 36.2, median: 28). Histologically, three cases were fibrotic tumors composed of spindle‐shaped cells with mild to moderate nuclear atypia and the collagenous stroma accompanied by woven bones or mature lamellar‐like bones. Two cases had cartilage formation. MDM2 and CDK4 expression was observed in two out of three cases on immunostaining. The histopathology of these three cases was regarded as the counterpart of low‐grade osteosarcomas, namely, parosteal osteosarcoma and low‐grade central osteosarcoma, arising in long bones.

**Conclusions:**

The surprisingly high incidence (60%, 3/5 cases) of low‐grade osteosarcoma explains the reason why gnathic osteosarcomas present a more favorable prognosis than osteosarcomas arising in long bones. Furthermore, it provides insight into the tumorigenesis mechanism of low‐grade osteosarcomas arising in the jaw and other sites.

## 1 INTRODUCTION

Primary osteosarcoma of the jaw bones accounts for approximately 10% of all osteosarcoma cases (Paparella et al., 2013; van den Berg et al., 2013). Previous studies have proposed that gnathic osteosarcomas occur in older ages compared to those affecting the long bones, showing favorable prognoses (Lee et al., 2015; Paparella et al., 2013). However, details regarding the histological feature of gnathic osteosarcoma are still obscured. Some studies have indicated that conventional osteosarcoma is the predominant histological type (Baumhoer et al., 2014; Bennett et al., 2000; Jasnau et al., 2008; Thariat et al., 2013; van den Berg et al., 2013). Other studies have shown a predominance of low‐grade histology (Lopes et al., 2001; Padilla & Murrah, 2011). Bennett et al. (2000) indicated that the majority of tumors showed focal chondroid formation. To clarify the specific clinicopathological features of gnathic osteosarcoma, we reviewed seven Japanese cases.

Conventional osteosarcoma generally arises in teenagers, and sites of predilection involve the metaphysis of the long bones, especially the distal femur and the proximal tibia. Microscopically, the neoplastic cells typically show severe atypia, producing varying amounts of neoplastic bone. Conventional osteosarcoma is subdivided into three histological patterns based on the neoplastic matrix components, namely, osteoblastic, chondroblastic, and fibroblastic (Rozeman et al., 2006; WHO Classification of Tumours Editorial Board, 2020). Moreover, there are four specific types of osteosarcoma that show peculiar histological and clinical features distinct from those of conventional osteosarcoma. Among these specific types, parosteal osteosarcoma and low‐grade central osteosarcoma are its low‐grade variants. Parosteal osteosarcoma arises on the surface of the bone, preferentially on the distal posterior femur followed by the proximal portions of the tibia and humerus. Histologically, this variant demonstrates spindle cell tumors with hypo‐ to moderate cellularity, forming variable bone and cartilage components, wherein woven bone and well‐formed trabeculae are frequently observed. Its peak incidence occurs in the third decade of life, with a slight female predominance. Prognosis is usually excellent, but approximately 8%–25% of the tumors show progression to the high‐grade osteogenic or non‐osteogenic sarcoma, with a prognosis similar to that of conventional osteosarcoma (Hang & Chen, 2014; Ruengwanichayakun et al., 2019). On genetic analysis of this variant, chromosome amplification of the region 12q13‐15, which contains MDM2 and CDK4 genes was observed in 85% of the cases (Yoshida et al., 2010). On the other hand, low‐grade central osteosarcoma has clinical and histological features similar to those of parosteal osteosarcoma, except that it arises within the medullary cavity of the bone (Schwab et al., 2008). Parosteal osteosarcoma accounts for 4%, while low‐grade central osteosarcoma accounts for 1%–3% of all osteosarcomas, respectively (Hang & Chen, 2014; Schwab et al., 2008).

In this study, we found that three out of a total of five cases who had gnathic primary osteosarcoma showed clinicopathological features such as low‐grade osteosarcomas. Investigating these cases may explain why gnathic osteosarcomas show a favorable prognosis as compared to long bone osteosarcomas.

## 2 MATERIALS AND METHODS

Diagnosed cases of jaw osteosarcoma, not otherwise specified of the jaw at Keio University Hospital from 2000 to 2016, were obtained from the laboratory information system. Both primary and secondary osteosarcomas were included, while metastasis from other sites (i.e., long bones) was excluded. Seven cases were obtained and reviewed by two pathologists listed among the authors (AS and KK). Clinical information was obtained from the hospital's information system, and histological assessment was performed on the following points: cellular atypia, osteoid formation, woven bone formation, cartilage formation, collagenous stroma formation, number of mitotic figures, and necrosis. Additionally, immunohistochemical studies were performed on sections obtained from formalin‐fixed, paraffin‐embedded tissues preserved at the Division of Diagnostic Pathology Laboratory using BOND III Automated Immunostainer (BOND III, Leica Biosystems, Wetzlar, Germany). The primary antibodies used in the study were CDK4 (clone DCS‐31, dilution 1: 200, Invitrogen, Carlsbad, CA, USA) and MDM2 (clone IF2, dilution 1:200, Thermo Fisher Scientific, Waltham, MA, USA), and a positive test was confirmed when approximately 30% of tumor cells showed significant nuclear staining compared to the internal negative control cells.

The study protocol was approved by the Keio University School of Medicine Ethics Committee (No. 20180087) and the Tokyo Dental College Ichikawa General Hospital Ethics Committee (No. I19‐01).

## 3 RESULTS

The clinical features of seven cases are summarized in Table 1. The study population comprised of one male and six females, of which two patients had secondary osteosarcomas—cases 5 and 6. Notably, case 5 had a history of radiation therapy for squamous cell carcinoma of the paranasal sinus 10 years prior to diagnosis of osteosarcoma, but we had no further information regarding the hereditary predisposition or genetic changes in this case. Case 6 was diagnosed with fibrous dysplasia of the maxilla from approximately 20 years prior, but GNAS mutation was not detected on cancer multi‐gene panel testing. The patients' ages ranged from 26 to 58 years (mean 41.9, median 42). As for five primary osteosarcoma cases, the age ranged from 26 to 58 years (mean: 36.2, median: 28). The maxilla and mandible were almost equally affected in these cases.

**TABLE 1:** Summary of clinical features

Case	Age	Sex	Site	Tx	PH	Met	Outcome
1	26	F	L/UJ	Res	None	None	ANED (10y)
2	27	F	L/LJ	CTx/Res	None	None	ANED (18y)
3	28	F	L/LJ	Res	None	None	N/A (3y)
4	42	F	L/UJ	CTx/Res	None	None	ANED (5y)
5	55	M	MS	N/A	RTx	N/A	N/A (1y)
6	57	F	R/UJ	HPTx	FD	None	D (4y)
7	58	F	R/LJ	CTx/Res	None	Lung	N/A (3y)

Abbreviations: ANED, alive no evidence of disease; CTx, chemotherapy; D, dead; F, female; FD, fibrous dysplasia; HPTx, heavy particle therapy; L, left; LJ, lower jaw; M, male; Met, metastasis; MS, maxillary sinus; N/A, not available; PH, past history; R, right; Res, resection; RTx, radiation therapy; Tx, treatment; UJ, upper jaw.

Radiographic images were obtained from three cases: cases 1, 4, and 6 (Figure S1). Computed tomography (CT) of case 1 prior to second time resection showed a mineralized tumor around the left upper jaw (Figure S1(a,b)). The patient had received initial resection of the tumor 6 years before the second resection, and the initial pathological diagnosis was a fibro‐osseous lesion. However, images obtained before the first resection were not available. Meanwhile, CT of case 4 showed a diffuse mass arising from the maxillary alveolar bone surface invading the pterygopalatine fossa (Figure S1(c,d)), and CT of case 6 showed a mineralized mass on the maxilla extending to the nasal cavity (Figure S1(e,f )).

**FIGURE 1:**
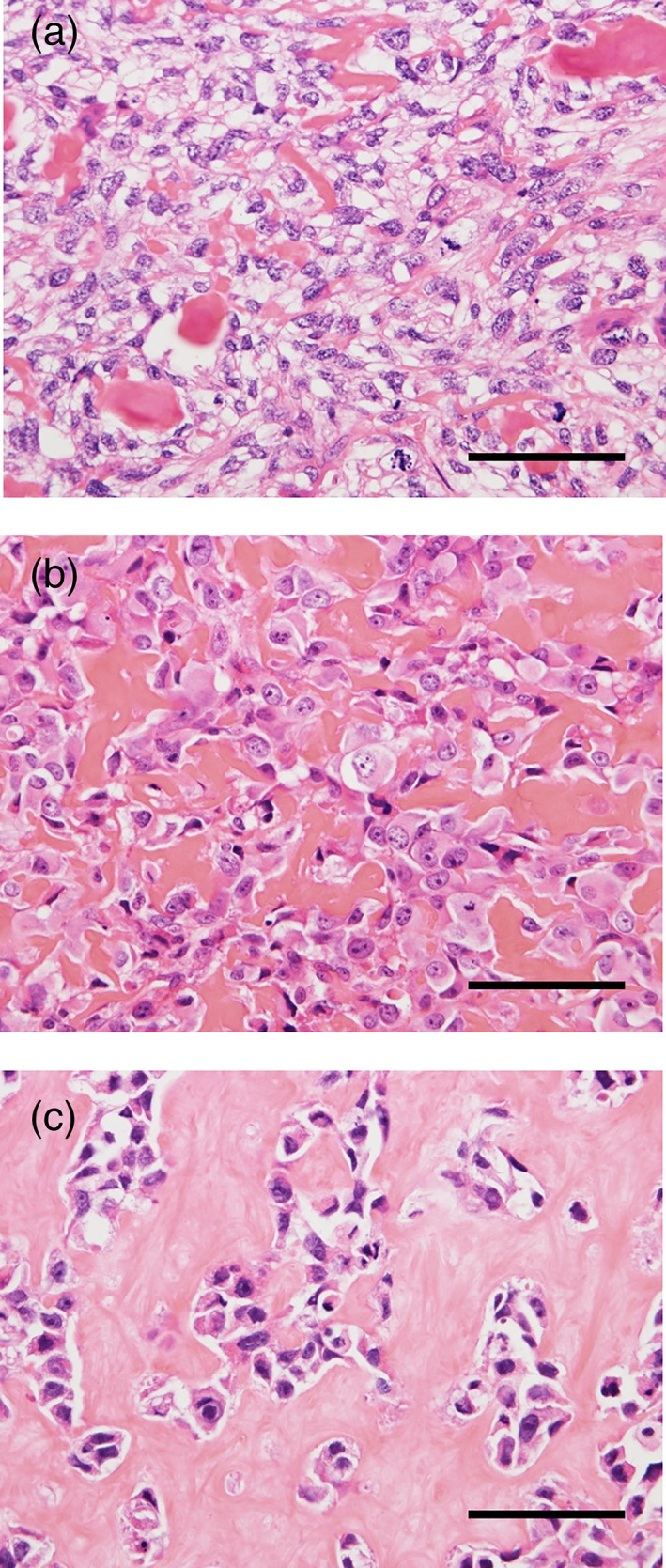
High‐grade conventional osteosarcomas showed in case 5 (a), case 5 (b), and case 3 (c). The scale bars indicate 50 μm

**FIGURE 2:**
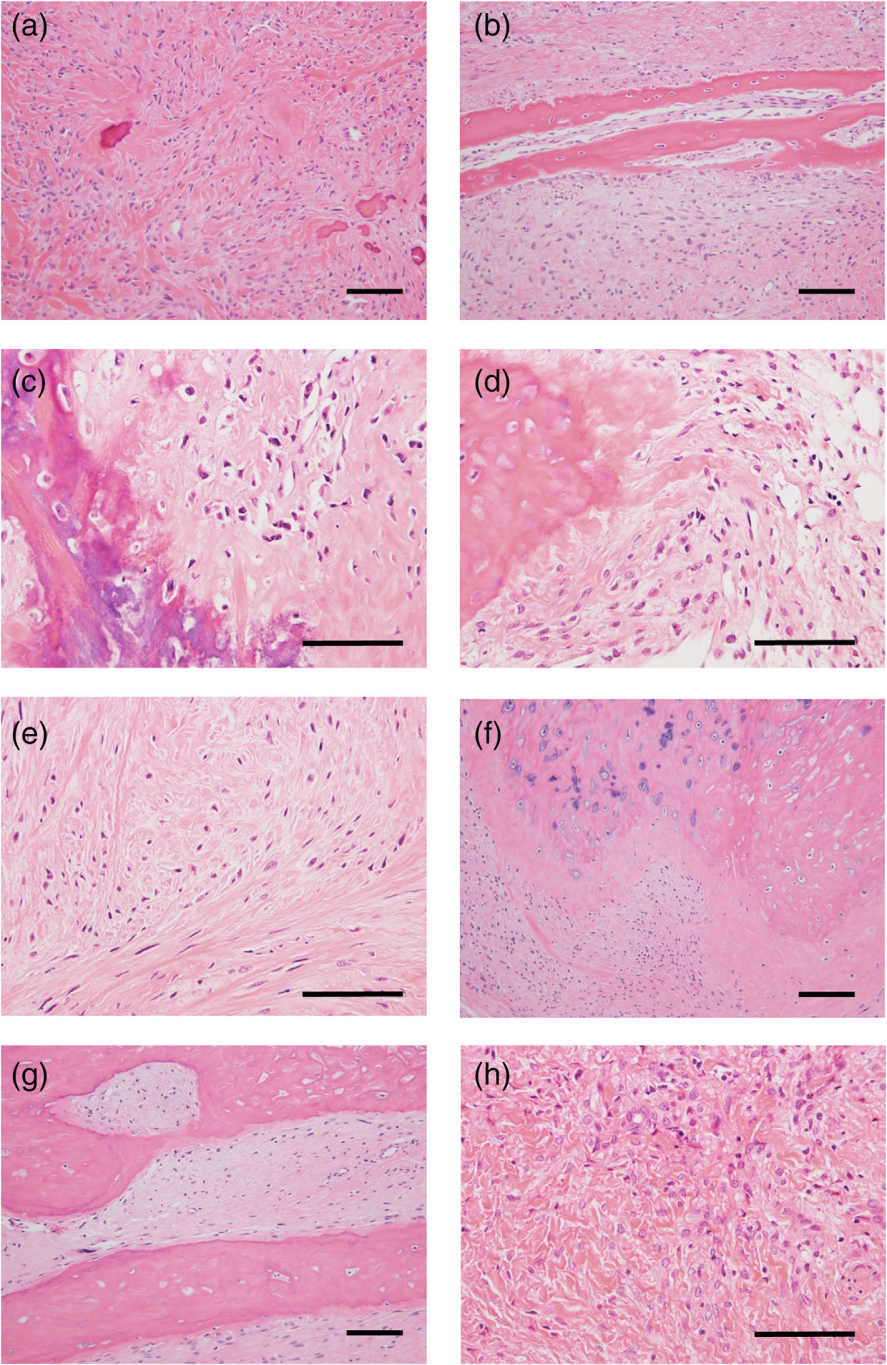
Low‐ and intermediate‐grade osteosarcomas showed in case 1 (a,b), case 2 (c,d), and case 4 (e–g). Low‐grade osteosarcoma component of case 7 (h). The scale bars indicate 50 μm

**FIGURE 3:**
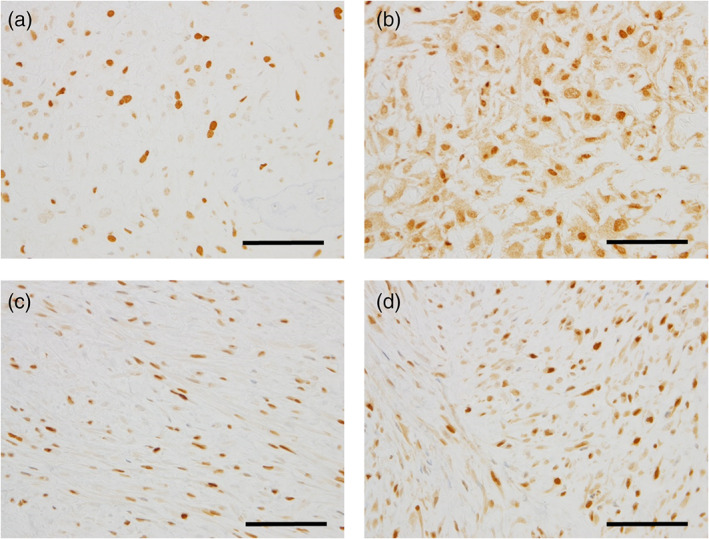
MDM2 and CDK4 immunostaining. (a) Case 1, MDM. (b) Case 1, CDK4. (c) Case 4, MDM2. (d) Case 4, CDK4. The scale bars indicate 50 μm

**FIGURE 4:**
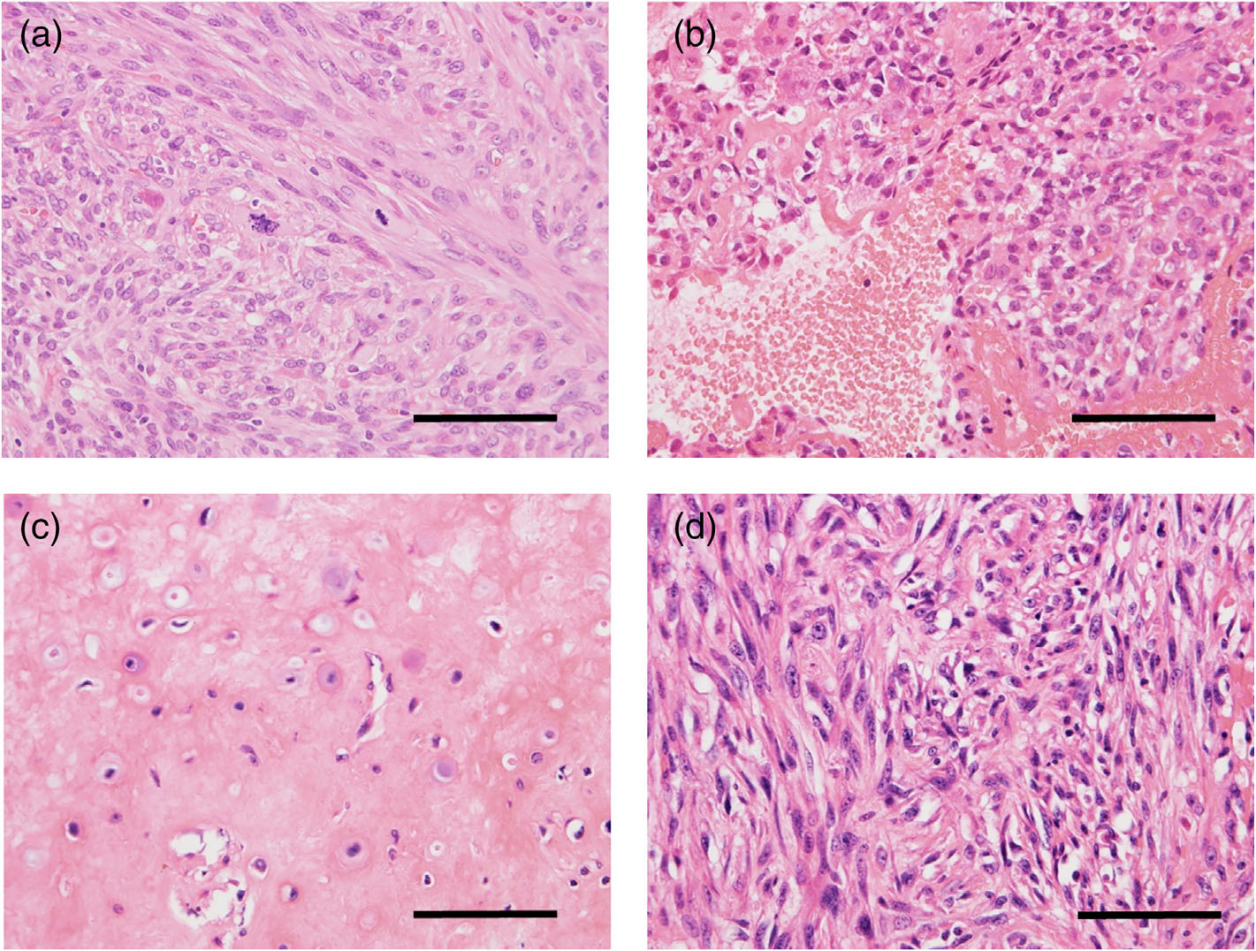
(a) Non‐osteogenic high‐grade sarcoma observed in case 4. (b) High‐grade osteosarcoma component of case 7. (c) Osteochondrogenic tumor observed in lung metastasis of case 7. (d) Non‐osteogenic tumor observed in lung metastasis of case 7. The scale bars indicate 50 μm

Histological evaluation was performed on biopsy specimens since the histology had been modified on resected specimens by preoperative chemotherapy. Microscopically, two secondary cases presented with high‐grade osteosarcoma with marked nuclear atypia and lace‐like osteoid formation, as typically observed in conventional osteoblastic osteosarcoma (Figure 1(a,b)). One out of five cases of primary osteosarcoma presented with high‐grade osteoblastic osteosarcoma (case 3) (Figure 1(c)). Meanwhile, the remaining four primary cases (cases 1, 2, 4, and 7) were fibrotic tumors composed of spindle‐shaped cells with mild to moderate nuclear atypia embedded in the collagenous stroma (Figure 2), and lace‐like osteoids were rarely observed. Moreover, woven bones or mature lamellar‐like bones were observed in cases 1 (Figure 2(b)), 2 (Figure 2(c,d)), and 4 (Figure 2(g)), and cartilages were observed in cases 2 and 4 (Figure 2(f)). The histological diagnosis of osteosarcoma at biopsy was very challenging in case 7 due to the specimen's small size, wherein most of the sections showed only moderate atypia (Figure 2(h)), and a small segment showing severe atypia (Figure 4(b)) led to the diagnosis of osteosarcoma. Additionally, mitotic figures were few in primary osteosarcomas, namely, cases 1, 2, 3, and 4, meanwhile mitotic figures were rarely observed in case 7; however, we could not quantify the number of mitotic figures in case 7 because the specimen was too small. Notably, tumor necrosis was observed only in case 5, the secondary osteosarcoma following radiation therapy. Furthermore, MDM2 and CDK4 expression were observed on immunostaining in cases 1 (Figure 3(a,b)), 4 (Figure 3(c,d)), and 7. The histological features of the biopsy specimens are summarized in Tables 2 and 3.

**TABLE 2:** Summary of histological features

Case	Atypia	Osteoid	WB	Cart	CF	Mitosis	Necrosis
1	M	±	+	−	++	3 per 50 HPF	−
2	M	±	+	+	+	0 per 50 HPF	−
3	M to H	+	+	−	−	2 per 50 HPF	−
4	L to M	−	+	+	++	1 per 50 HPF	−
5	H	+	+	−	+	66 per 50 HPF	+
6	H	++	±	−	+	17 per 50 HPF	−
7	M to H	±	±	−	++	n.d.	−

*Note*: The symbols indicate as the following: (++), markedly positive; (+), positive; (±), positive but very minor component; (−), negative.

Abbreviations: Cart, cartilage; CF, collagen fiber; H, high; HPF, high‐power field; L, low; M, moderate; WB, woven bone.

**TABLE 3:** Summary of immunostaining

Case	MDM2	CDK4
1	+	+
2	−	−
3	±	±
4	+	+
5	±	−
6	+, focal	+, focal
7	+	+

*Note*: The symbols indicate as the following: (+), positive throughout the tumor; (±), positive but weak; (−), negative. The words (+, focal) indicate that the positive area is focal.

Case 4 developed into a high‐grade, non‐osteogenic tumor (dedifferentiation) 4 years after the first presentation to our hospital (Figure 4(a)), and the details of this case have been described previously (Miyashita et al., 2018). Case 7 presented with lung metastasis 2 years after the first presentation, in which histology of the metastasis was composed of a mixture of osteochondrogenic (Figure 4(c)) and non‐osteogenic (Figure 4(d)) tumors.

For treatment, two patients underwent simple resection (cases 1 and 3), and three underwent resection after chemotherapy (cases 2, 4, and 7). The therapeutic effects of chemotherapy showed a complete response in case 7 and a partial response in cases 2 and 4, respectively. However, the outcomes of cases 3, 5, and 7 were not available because they were transferred to another hospital. Other cases were alive, and the maximum observation period was 16 years. The observation periods are shown in parenthesis in Table 1.

## 4 DISCUSSION

In this study, five cases of primary osteosarcoma and two cases of secondary osteosarcoma were assessed. The two cases of secondary osteosarcoma (cases 5 and 6) occurred in the 50th decade of life and presented with a high‐grade osteosarcoma comparable to that of the conventional osteosarcoma. The features were identical to those of the secondary osteosarcoma that arises in the other bone sites. As for cases of primary osteosarcoma, the mean age was 36 years, and there was a female predominance. On the other hand, the histology was quite different from that of conventional osteosarcoma in three cases (cases 1, 2, and 4), presenting a fibrotic tumor with mild to moderate cellular atypia. Furthermore, two out of these three (cases 1 and 4) showed nuclear immunoreactivity to MDM2 and CDK4, indicating their compatibility with low‐grade osteosarcoma. Case 2 also seemed to morphologically has low‐grade osteosarcoma features, although MDM2 and CDK4 were not expressed immunohistochemically. The absence of MDM2 and CDK4 expression could be ascribed to the fixation and decalcification difficulties since case 2 was the oldest in this study. Case 7 presented with a conventional high‐grade osteosarcoma, however, the presence of low‐grade osteosarcoma components (Figure 2(h)) and the MDM2 and CDK4 immunoreactivity suggests that the patient had progressed from low‐grade osteosarcoma. As mentioned previously, low‐grade osteosarcomas are divided into parosteal osteosarcoma and low‐grade central osteosarcoma based on the sites of involvement. Case 1 macroscopically showed an extra‐ and intramedullary mass macroscopically during the first resection (data not shown), and the case 4 presented a diffuse radiopaque lesion around the left molar region on CT imaging (Miyashita et al., 2018), indicating that both cases had parosteal osteosarcoma. It is difficult to determine between parosteal osteosarcoma and low‐grade central osteosarcoma in cases 2 and 7 due to the unavailability of initial imaging or macroscopic pictures.

Chondroid differentiation has been identified in approximately half of the cases of parosteal osteosarcoma (Hang & Chen, 2014; WHO Classification of Tumours Editorial Board, 2020). In our cases, two out of three low‐grade osteosarcomas contain cartilage. Although there is no significant difference in survival between the histological subtypes of conventional osteosarcoma, it has been suggested that chondroblastic tumors have a slightly better survival despite poor response to chemotherapy (Hauben et al., 2002). These results indicate that chondroid differentiation may be regarded as a well‐differentiated phenotype of osteosarcomas.

It is difficult to assess the prognosis of gnathic osteosarcomas in our cases since the observation period was less than 5 years in most cases. However, it is remarkable that case 1 had been alive for 10 years with no evidence of disease, even though she had not undergone chemotherapy.

As for long bones, low‐grade osteosarcomas account for 5%–6% of all osteosarcomas (Hang & Chen, 2014; Schwab et al., 2008). In our cases, 60% (3 out of 5 cases) of gnathic primary osteosarcomas presented with low‐grade osteosarcoma, which is a significantly high rate. Although our study has limitations due to its small sample size and short observation period, the results may infer why gnathic osteosarcomas show a favorable prognosis as compared to osteosarcomas arising in long bones.

In some previous reports, low‐grade osteosarcomas were not predominant as compared to conventional osteosarcomas in gnathic cases (Baumhoer et al., 2014; Bennett et al., 2000; Jasnau et al., 2008; Thariat et al., 2013; van den Berg et al., 2013). This discrepancy with our findings may be attributed to not only the difference in sample size, but also racial differences since the above‐cited reports were all from European ethnicity. Another reason for this disparity could be the limited observation period for each study. In the past, when low‐grade osteosarcomas were overlooked by clinicians and pathologists, it could be misdiagnosed as a benign disease, such as fibrous dysplasia (Tabatabaei et al., 2015). Therefore, a study that constitutes a large number of old cases from the 1960s and the 1970s may show no predominance of low‐grade osteosarcomas.

The rationale behind the high incidence of low‐grade osteosarcomas in gnathic osteosarcomas is unclear. We speculate that the osteogenesis peculiar to jawbones may be related to the pathogenesis of low‐grade osteosarcomas. There are two known pathways of osteogenesis: endochondral ossification and intramembranous ossification (Long & Ornitz, 2013; Su et al., 2018). In endochondral ossification, the cartilaginous mold is first formed from the mesenchymal cells. The mineralized cartilage is then replaced with bone matrix produced by osteoblasts derived from the periosteum. This ossification process occurs at the ossification center, situated in the metaphysis (Tabatabaei et al., 2015). However, in intramembranous ossification, however, mesenchymal cells directly differentiate into osteoblasts and form the bone matrix without prior cartilage formation (Runyan & Gabrick, 2017). Most of the bones, including the long bones of the limbs, undergo endochondral ossification. In contrast, the craniofacial bones, including the maxilla and mandible, mainly undergo intramembranous ossification during osteogenesis (Runyan & Gabrick, 2017). Intramembranous ossification also occurs at the surface of long bones or in the medullary cavity during the remodeling phase (Frohlich et al., 2008). Although the etiology of osteosarcoma is still unknown, several studies, using in vivo assays or mouse model systems have suggested that growth factors and signaling cascades working on the growth plate play roles in the development of osteosarcoma (He et al., 2017; Rubio et al., 2014). This fact leads to the speculation that the disturbance of the growth signals during endochondral ossification may be an essential cause of conventional osteosarcoma. As for jawbones, which mainly undergo intramembranous ossification, the different oncogenic signals may play a role in the tumorigenesis of osteosarcoma. It is remarkable that both parosteal osteosarcoma and low‐grade central osteosarcoma of the long bones both seem to arise at sites and in ages that usually do not present with endochondral ossification. Therefore, it is speculated that oncogenic signals of low‐grade osteosarcomas arising both in the jaws and long bones are closely related to the growing signals of intramembranous ossification, which is different from that of endochondral ossification. For example, platelet‐derived growth factors (PDGFs) and PDGF receptors (PDGFRs), which promote the migration of mesenchymal stem cells at intramembranous ossification sites, are variably expressed in osteosarcomas (Shah et al., 2014; Xu et al., 2018). Interestingly, PDFGR signaling promotes tumorigenesis in concert with CDK4 in a murine glioma model (Hoeman et al., 2018). It has been suggested that MDM2 and the PI3K/AKT/mTOR pathway, which is one of the downstream PDGFR pathways, work synergistically (Laroche et al., 2017). Therefore, the MDM2 and CDK4, usually amplified in low‐grade osteosarcomas, might be key molecules involved in the tumorigenesis of low‐grade osteosarcomas. The tumorigenesis mechanism of osteosarcomas, both conventional and low‐grade, gnathic, and other sites must be elucidated in further investigations.

## 5 CONCLUSION

We found three out of five cases of gnathic primary osteosarcomas which showed clinicopathological features resembling those of low‐grade osteosarcomas. The surprisingly high incidence (60%, 3/5 cases) of low‐grade osteosarcoma may explain the reason why gnathic osteosarcomas present a more favorable prognosis compared to osteosarcomas arising in long bones. Furthermore, it postulates that the tumorigenesis mechanism of low‐grade osteosarcomas arising in the jaw and the other sites might be closely related to intramembranous ossification.

## CONFLICT OF INTEREST

The authors have no conflict of interest to declare.

## Supporting information


**Figure S1 :** CT images of case 1 (a,b), case 4 (c,d), and case 6 (e,f )

## Data Availability

The data that support the findings of this study are available on request from the corresponding author. The data are not publicly available due to privacy or ethical restrictions.
