# Supplementary figures and images for: Low‐grade osteosarcoma is predominant in gnathic osteosarcomas: A report of seven cases of osteosarcoma of the jaw
Source: Clin Exp Dent Res. 2021 May 19;7(6):1175–82. doi: 10.1002/cre2.442 (PMC8638322; doi:10.1002/cre2.442)

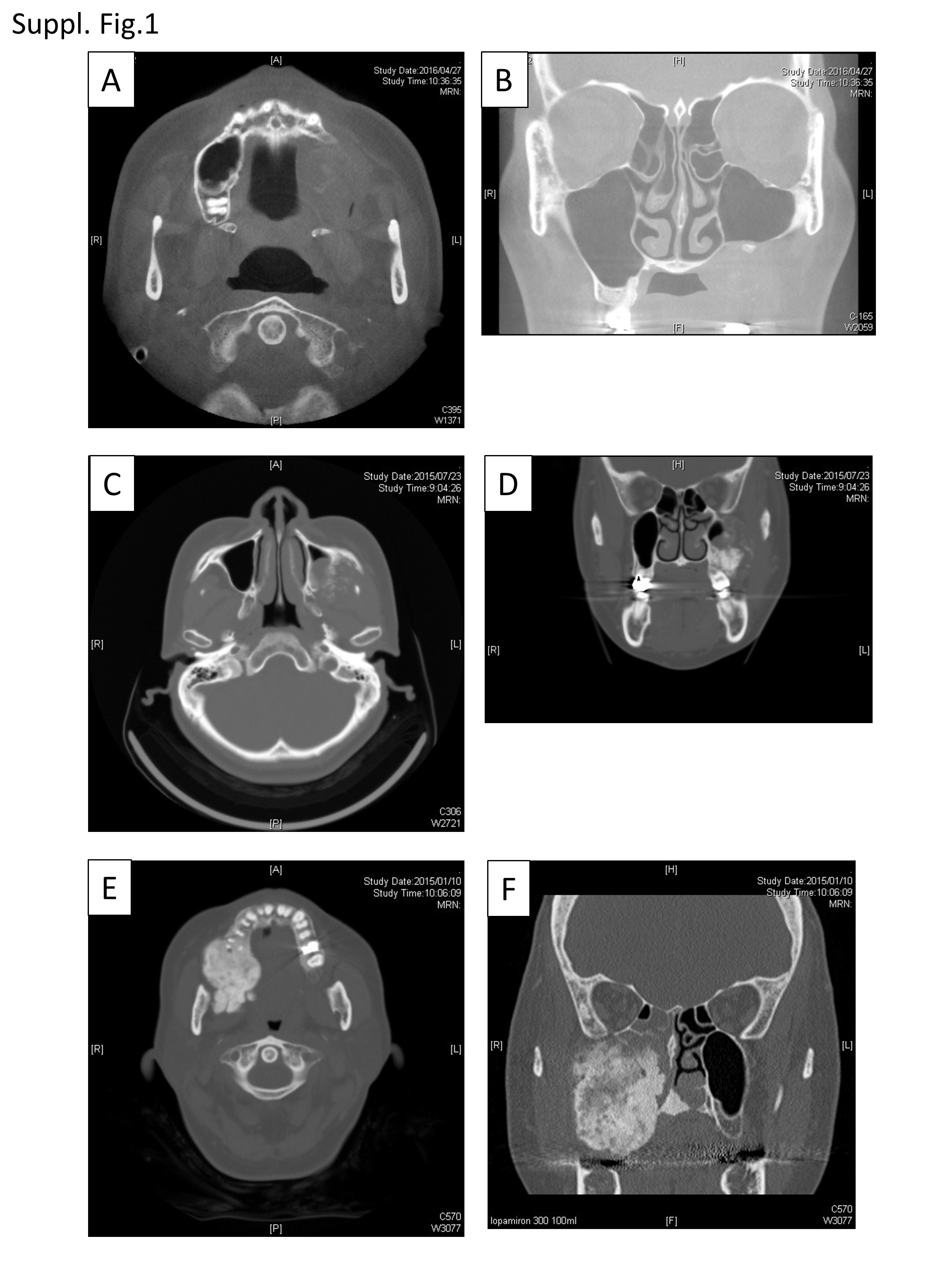

Supplement: Supplementary file 1 — Figure S1 : CT images of case 1 (a,b), case 4 (c,d), and case 6 (e,f ) [file CRE2-7-1175-s001.TIF]
